# Summer temperature increase has distinct effects on the ectomycorrhizal fungal communities of moist tussock and dry tundra in Arctic Alaska

**DOI:** 10.1111/gcb.12716

**Published:** 2014-10-08

**Authors:** Luis N Morgado, Tatiana A Semenova, Jeffrey M Welker, Marilyn D Walker, Erik Smets, József Geml

**Affiliations:** 1 Naturalis Biodiversity CenterP.O. Box 9517, Leiden, RA, 2300, The Netherlands; 2 Faculty of Science, Leiden UniversityP.O. Box 9502, Leiden, RA, 2300, The Netherlands; 3 Department of Biological Sciences, University of Alaska AnchorageAnchorage, USA; 4 HOMER Energy1790 30th St, Suite 100, Boulder, CO, 80301, USA; 5 Plant Conservation and Population Biology, KU LeuvenKasteelpark Arenberg 31, Box 2437, Leuven, 3001, Belgium

**Keywords:** arctic ecology, climate changes, fungal ecology, fungi, ITEX, long-term ecological research, Toolik Lake

## Abstract

Arctic regions are experiencing the greatest rates of climate warming on the planet and marked changes have already been observed in terrestrial arctic ecosystems. While most studies have focused on the effects of warming on arctic vegetation and nutrient cycling, little is known about how belowground communities, such as fungi root-associated, respond to warming. Here, we investigate how long-term summer warming affects ectomycorrhizal (ECM) fungal communities. We used Ion Torrent sequencing of the rDNA internal transcribed spacer 2 (ITS2) region to compare ECM fungal communities in plots with and without long-term experimental warming in both dry and moist tussock tundra. *Cortinarius* was the most OTU-rich genus in the moist tundra, while the most diverse genus in the dry tundra was *Tomentella*. On the diversity level, in the moist tundra we found significant differences in community composition, and a sharp decrease in the richness of ECM fungi due to warming. On the functional level, our results indicate that warming induces shifts in the extramatrical properties of the communities, where the species with medium-distance exploration type seem to be favored with potential implications for the mobilization of different nutrient pools in the soil. In the dry tundra, neither community richness nor community composition was significantly altered by warming, similar to what had been observed in ECM host plants. There was, however, a marginally significant increase in OTUs identified as ECM fungi with the medium-distance exploration type in the warmed plots. Linking our findings of decreasing richness with previous results of increasing ECM fungal biomass suggests that certain ECM species are favored by warming and may become more abundant, while many other species may go locally extinct due to direct or indirect effects of warming. Such compositional shifts in the community might affect nutrient cycling and soil organic C storage.

## Introduction

Soils of the northern circumpolar region cover approximately 16% of the global soil surface and contain an estimated 50% of all soil organic carbon (C) pool (Tarnocai *et al*., 2009). Because these regions have been experiencing some of the highest rates of warming (0.06–0.1 °C per year over the past 40 years), a large proportion of this C is increasingly vulnerable to mobilization due to warming-induced melting of permafrost and higher microbial decomposition rates (Anisimov *et al*., 2007; Hansen *et al*., 2010; Comiso x0026; Hall, 2014). This warming is resulting in a suite of climate feedbacks, including changes in sea ice cover and the length of ice-free periods (Arrigo x0026; van Dijken, 2011; Post *et al*., 2013), a greening of the surrounding land surface, and tree line advancement (Kharuk *et al*., 2013; Zhang *et al*., 2013). All of these are altering the albedo of the Arctic (Chapin *et al*., 2005; Post *et al*., 2009). Although many of these feedbacks are positive, some could potentially be negative. For example, a greening of the Arctic driven by increases in shrub density (Sturm *et al*., 2005; Loranty x0026; Goetz, 2012; Tape *et al*., 2012) could result in greater degrees of C sequestration (Welker *et al*., 1997; Sistla *et al*., 2013; Anderson-Smith 2013; Pattison x0026; Welker, 2014), but see Hartley *et al*. (2012) for counterargument. Increases in shrub density and canopy growth can further alter the tundra by local snow trapping in winter, increasing soil insulation, causing higher winter, and spring-time soil temperatures, and increasing the rates of nitrogen (N) and C mineralization. Greater rates of winter CO_2_ emissions, in turn, may enhance the potential for shrub growth and further expansion (Sturm *et al*., 2001; Schimel *et al*., 2004; Sturm *et al*., 2005; Weintraub x0026; Schimel, 2005; Tape *et al*., 2006). However, whether these changes are accompanied by a simultaneous reorganization of the soil fungal community and whether these responses differ in moist tussock and dry tundra have not been resolved.

Arctic soils have limited availability of nutrients and arctic plants are highly dependent on mutualistic relationships with mycorrhizal fungi for survival (Gardes x0026; Dahlberg, 1996; Hobbie *et al*., 2009; Bjorbækmo *et al*., 2010). It has been estimated that 61–86% of the N in Arctic tundra plants is obtained through mycorrhizal fungi (Hobbie x0026; Hobbie, 2006). Ectomycorrhizal (ECM) fungi are the predominant fungal guild in the Arctic (Gardes x0026; Dahlberg, 1996; Clemmensen *et al*., 2006; Bjorbækmo *et al*., 2010). Recent studies of belowground Arctic ECM fungal communities, revealed higher species richness than what had previously been known from aboveground surveys (Ryberg *et al*., 2009; Bjorbækmo *et al*., 2010; Geml *et al*., 2012; Timling *et al*., 2012; Timling x0026; Taylor, 2012). These studies indicated that the most diverse arctic ECM genera are *Tomentella* (here interpreted as including *Thelephora)*, *Inocybe*, *Cortinarius*, *Sebacina*, *Russula,* and *Hebeloma*.

ECM fungal community composition in the Arctic is generally correlated with soil properties, geology, plant productivity, and climate (Timling *et al*., 2012, 2014). There is also evidence to suggest that ECM plant host identity is not a main driver of ECM fungal community composition in the Arctic (Ryberg *et al*., 2009; Timling *et al*., 2012). Although there are a few studies focused on the molecular diversity of belowground ECM fungal communities in the Arctic (Ryberg *et al*., 2009; Bjorbækmo *et al*., 2010; Geml *et al*., 2012 Timling *et al*., 2012, 2014), the main drivers at the landscape scale remain largely unresolved, and this hampers our current in-depth comprehension of arctic soil ecology.

Recent evidences, reported from other biomes than the Arctic, suggest that the extramatrical mycelium (EMM) morphology and ECM fungi extracellular enzyme activity are of great relevance to understand the nutrient dynamics of the ECM symbiosis (Cairney x0026; Burke, 1996; Agerer, 2001; Anderson x0026; Cairney, 2007; Hobbie x0026; Agerer, 2010; Peay *et al*., 2011; Tedersoo *et al*., 2012; Talbot *et al*., 2013; Bödeker *et al*., 2014) that is crucial to understand soil ecology. ECM fungi produce EMM that grows from the ectomycorrhizae into the surrounding soil with the crucial functions of foraging the litter and/or mineral layers for nutrients, and of seeking new root tips for colonization (Martin *et al*., 2001; Anderson x0026; Cairney, 2007). The EMM forms an intricate hyphal network that interconnects plant roots, and paves the way for interplant C and nutrient movements (Selosse *et al*., 2006). EMM of different taxa are known to have distinct anatomical and physiological features that are attributable to various strategies of foraging (Colpaert *et al*., 1992; Agerer, 2001; Hobbie x0026; Agerer, 2010). Several studies linked the EMM characteristics with the pools of nutrients they explore in the soil, and with their roles in soil-plant interaction, taking into account energetic cost-benefit for both fungi and plant host (e.g., Agerer, 2001; Lilleskov *et al*., 2002; Hobbie x0026; Agerer, 2010; Lilleskov *et al*., 2011; Cairney, 2012). The main characteristics to classify the EMM are the mycelium exploration type, presence/absence of rhizomorphs and hydrophobicity of the hyphae (Agerer, 2001; Hobbie x0026; Agerer, 2010; Peay *et al*., 2011; Lilleskov *et al*., 2011; Cairney, 2012). Moreover, besides EMM characteristics *per se*, species with abundant EMM generally showed stronger potential to produce extracellular enzymes than species with scarce EMM (Tedersoo *et al*., 2012), even though multiple exceptions exist. It has been hypothesized that species with EMM of the medium-distance fringe, and long-distance exploration types might have the potential to explore recalcitrant nutrient pools through extracellular enzyme activity, and that species with contact, short, and medium-distance smooth exploration types might be associated with labile nutrient soil pools (e.g., Lilleskov *et al*., 2002; Hobbie x0026; Agerer, 2010; Lilleskov *et al*., 2011). Such functional information is still under investigation, and therefore, currently only available for a limited number of taxa. Nevertheless, this framework constitutes a valuable insight into the ecological functions of ECM fungal community.

The long-term effects of climate change on arctic tundra function and structure have primarily been investigated with respect to aboveground growth, phenology, vegetation composition, and plant and ecosystem C exchange (e.g., Chapin x0026; Shaver, 1985; Arft *et al*., 1999; Welker *et al*., 1997, 2000, 2004; Elmendorf *et al*., 2012; Tape *et al*., 2012; Cahoon *et al*., 2012; Sharp *et al*., 2013; Pattison x0026; Welker, 2014). Vegetation studies, in the moist tussock tundra at Toolik Lake, Alaska, indicated that long-term experimental summer warming induced significant increases in the abundance and height of *Betula nana*, *Salix pulchra*, and graminoids, and in the accumulation of the litter layer (Wahren *et al*., 2005; Mercado-Díaz, 2011). Conversely, the bryophytes decreased significantly (Mercado-Díaz, 2011), most likely due to competitive exclusion by shrubs (Cornelissen *et al*., 2001; Jägerbrand *et al*., 2009). These aboveground vegetation changes are likely correlated with changes below ground, such as soil moisture, soil nutrient pools, fine-root abundance, and root turn-over dynamics, which interplay with ECM fungal community dynamics (e.g., Read *et al*., 2004; Dickie x0026; Reich, 2005; Dickie *et al*., 2005; Strand *et al*., 2008; Toljander *et al*., 2006; Twieg *et al*., 2009; Peay *et al*., 2011). Even though some studies addressed belowground processes, such as N cycling (e.g., Schimel *et al*., 2004; Borner *et al*., 2008; Schaeffer *et al*., 2013) and microbial community change (e.g., Clemmensen *et al*., 2006; Campbell *et al*., 2010; Deslippe *et al*., 2011, 2012), our knowledge about the compositional and functional changes of arctic communities in response to long-term warming remains rudimentary.

In this study, we use high-throughput sequencing techniques to study the long-term effects of experimental warming on the ECM basidiomycete community in dry and moist tussock tundra in Northern Alaska. Our hypotheses were twofold. First, we hypothesize that long-term warming induces changes in the ECM fungal community composition, because aboveground changes in the vegetation, including several ECM host plants, have already been documented (Wahren *et al*., 2005; Mercado-Díaz, 2011) and this is suggestive of changes in belowground processes (Sullivan x0026; Welker, 2005; Sullivan *et al*., 2007). Secondly, based on the results from the above vegetation studies and reported warming-induced increases in ECM fungal and fine-root biomass (Clemmensen *et al*., 2006), we expect that the ECM fungal community of the moist tussock tundra will show a stronger response to warming than the dry tundra. Furthermore, we expect to find a more diverse ECM community in the warmed moist tussock tundra plots, because Deslippe *et al*. (2011) reported significant increases in the diversity of arctic ECM fungi associated with root tips of *Betula nana* as a response to warming. *Betula nana* is a dominant in our sampling plots and has shown strong, positive response to experimental warming (Wahren *et al*., 2005; Mercado-Díaz, 2011).

## Material and methods

### Sampling location

The sampling area is located at the Arctic Long Term Ecological Research site in the Toolik Lake region in the northern foothills of the Brooks Range, Alaska, USA (68°37′N, 149°32′W; 760 m above sea level). The region lies within the bioclimatic subzone E that is the warmest subzone of the arctic tundra with mean July temperatures ranging from 9 to 12 °C (Walker *et al*., 2005). The two main vegetation types of the region are: the dry heath tundra, characterized by *Dryas octopetala*, *Salix polaris*, *Vaccinium* spp. and fruticose-lichens, and the moist tussock tundra, dominated by *Betula nana*, *Salix pulchra* and the sedge *Eriophorum vaginatum*. Detailed descriptions of the plant communities can be found in Walker *et al*. (1999) and Kade *et al*. (2005).

### Experimental design

Between July 23 and 25, 2012, we sampled soil from 20 plots representing the dry and the moist tussock tundra. In each tundra type, we sampled five plots that were subjected to passively increased summer air temperature by hexagonal open top chambers (OTCs), subsequently referred to as ‘treatment’, and five adjacent areas with unaltered conditions (‘control’). The sampling was performed with a soil corer of approximately 2 cm × 20 cm (diameter × depth). In each of the 20 plots, five soil cores were taken, thoroughly mixed and kept frozen until lyophilization.

The OTCs used are 1 m^2^, 0.4 m high, and constructed of translucent fiberglass (Marion *et al*., 1997; Walker *et al*., 1999). Within the OTCs the summer air temperature increases by a mean daily average of 1.5 °C, while soil temperatures remain the same as in the control plots (Walker *et al*., 1999). Every year, since 1994, the OTCs are set up as soon as 50% of the ground area of a given plot was snow free (usually early June) and are removed at the end of August or early September, following the International Tundra Experiment (ITEX) protocol (Welker *et al*., 1997). It has been repeatedly shown that OTCs provide a reasonable approximation to the predicted climatic changes in the Arctic as they alter daytime temperature significantly and minimize unwanted ecological effects, such as changes in soil moisture, the influence of wind speed on air temperature (Marion *et al*., 1997; Sharkhuu *et al*., 2013; Bokhorst *et al*., 2013 and references therein). Therefore, OTCs have been recommended to study the response of high-latitude ecosystems to warming (Marion *et al*., 1997).

### Molecular work

Genomic DNA was extracted from 1 ml (0.4–1 g) of lyophilized soil from each of the twenty samples using NucleoSpin® soil kit (Macherey-Nagel Gmbh x0026; Co., Düren, Germany), according to manufacturer's protocol. For each sample, two independent DNA extractions were carried out and pooled to optimize the homogenization of the extraction. The extracted DNA was eluted with 30 μl of SE buffer. PCR amplification and Ion Torrent sequencing of the ITS2 region (ca. 250 bp) of the nuclear ribosomal rDNA repeat were carried out as described by Geml *et al*. (2014b) using primers fITS7 (Ihrmark *et al*., 2012) and ITS4 (White *et al*., 1990). The ITS4 primer was labeled with sample-specific Multiplex Identification DNA-tags (MIDs). The amplicon library was sequenced using an Ion 318™ Chip by an Ion Torrent Personal Genome Machine (PGM; Life Technologies, Guilford, CT, USA) at the Naturalis Biodiversity Center.

The initial clean-up of the raw sequence data was carried out using the online platform Galaxy (https://main.g2.bx.psu.edu/root), in which the sequences were sorted according to samples and sequence regions of primers and adapters (identification tags) were removed. We used a parallel version of MOTHUR v. 1.32.1 (Schloss *et al*., 2009) for subsequent sequence analyses following the protocol described in detail in Geml *et al*. (2014b). The quality-filtered sequences were normalized following Gihring *et al*. (2012) by random subsampling, so that each sample contained 56 483 reads (the lowest number of sequences obtained for a sample). The resulting sequences were clustered into operational taxonomic units (OTUs) using OTUPIPE (Edgar, 2010) with the simultaneous removal of putatively chimeric sequences using *de novo* and reference-based filtering using curated dataset of fungal ITS sequences of Nilsson *et al*. (2011) as reference. We used a 97% sequence similarity clustering threshold as has been routinely done in fungal ecology studies (e.g., O'Brien *et al*., 2005; Higgins *et al*., 2007; Geml *et al*., 2008, 2009; Amend *et al*., 2010; Tedersoo *et al*., 2010; Geml *et al*., 2012; Kauserud *et al*., 2012; Brown *et al*., 2013; Blaalid *et al*., 2013; Geml *et al*., 2014a). Global singletons were discarded from further analysis. The reference database published by Kõljalg *et al*. (2013) was used to determine the taxonomic affinity of the OTUs using USEARCH v7 (Edgar, 2010). OTUs with less than 80% similarity to any identified fungal sequence were also excluded from the final analysis due to unreliable classification, and therefore, uncertainty regarding their ecological role. A representative sequence of each OTU was deposited in GenBank under the accession numbers KJ792472 – KJ792742.

### ECM fungal database and EMM determination

For in-depth analyses related to the research hypotheses stated above, we selected all OTUs that showed affinity with ECM basidiomycete genera based on Tedersoo x0026; Smith (2013). However, in Sebacinales, we used phylogenetic analyses to select the OTUs representing the ECM lineages, because many sebacinoid taxa are not ECM. In the Sebacinales, ECM OTUs were selected based on their supported phylogenetic placement (with ≥70% bootstrap and/or ≥0.95 posterior probability) among sequences of known ECM taxa published by Urban *et al*. (2003), Ryberg *et al*. (2009) and Tedersoo x0026; Smith (2013). We followed the work of Agerer (2006) and consulted the DEEMY database (http://deemy.de), an information system for the characterization and determination of ECM fungi (accessed in January and February of 2014), to determine the EMM characteristics per species. In the genus *Russula*, if no EMM information was available for the species of interest, we assumed the EMM characteristics based on the closest species with known characteristics. To determine the closest species we followed the phylogenetic study by Miller x0026; Buyck (2002). Similarly, for OTUs of the genus *Hebeloma*, we followed the phylogenetic study by Boyle *et al*. (2006).

### Statistical analysis

For each sample, we calculated rarefied OTU accumulation curves using the R package Vegan (Oksanen *et al*., 2012) and determined the Good's coverage (complement of the ratio between the number of local singletons and the total sequence counts). Because of demonstrated uncertainties regarding the reliability of read abundance as indicators of species abundance in the samples (Amend *et al*., 2010), we carried out the further analyses with two types of data transformations. First, we transformed the data into presence-absence matrix, where OTU presence was defined as five or more sequences on a per sample basis following the suggestion of Lindahl *et al*. (2013) to minimize false positives (e.g., OTUs that are common in one sample, but may be low-abundant contaminants in others). In addition, we used square-root transformed read abundance to moderate the influence of OTUs with high sequence counts, while maintaining some approximation of template abundance that may reflect ecological significance. We used PC-Ord v. 5.32 (McCune x0026; Grace, 2002) to run nonmetric multidimensional scaling (NMDS) on a primary matrix of experimental plots by OTUs and a secondary matrix of plots by OTU richness per taxon (this analysis was also performed with root-square abundance of sequence counts as a surrogate to species abundance). The dataset was subjected to 500 iterations per run using the Sørensen similarity (Bray-Curtis index) and a random starting number. We also calculated the Pearson's correlation coefficient (*r*) values between relative OTU richness, OTU diversity per taxon, and axes 1 and 2. We tested whether fungal communities were statistically different across the treatments using a multi-response permutation procedure (MRPP) and determined any preferences of individual OTUs for either control or treatment plots in moist tussock and dry tundra using Indicator Species Analyses (Dufrêne x0026; Legendre, 1997) as implemented in PC-Ord v. 5.32. We also tested for significant differences in OTU richness between moist tussock and dry tundra, control and treatment plots, genera, and EMM characteristics using Students *t*-test. We determined the Venn diagram for the genera with higher OTU richness, using the online version of the publication by Oliveros (2007).

## Results

### Taxonomic composition and OTU richness

**Fig 1:**
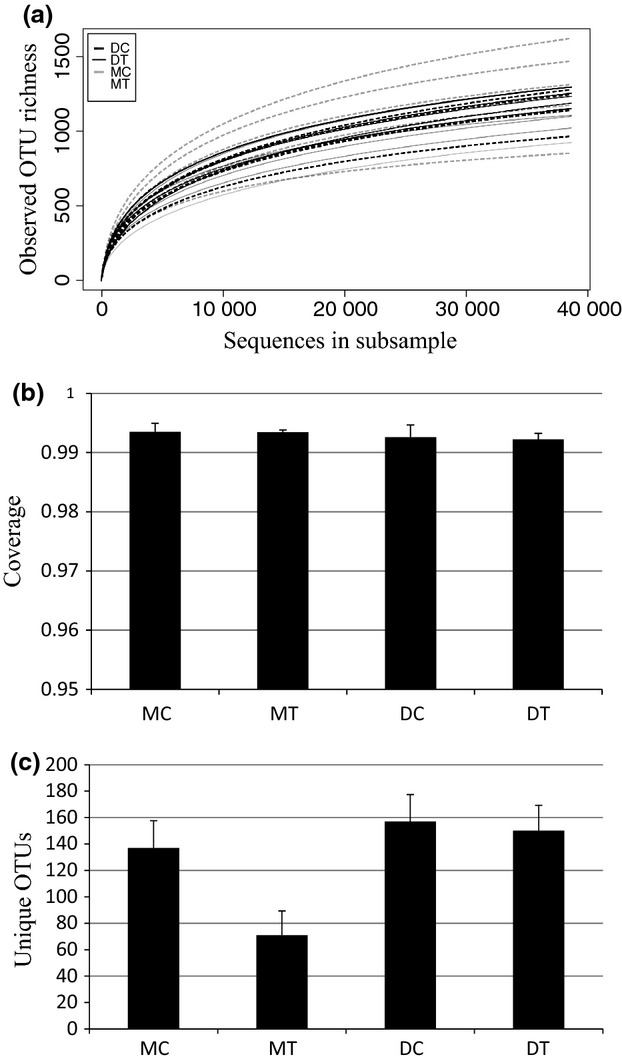
(a) Rarefaction curves of each plot for both tundra types. (b) Good's coverage, average of the plot per site with standard deviation. (c) Total OTUs per site and tundra type with standard deviation. DC, dry control; DT, dry warming treatment; MC, moist tussock control; MT, moist tussock warming treatment.

We obtained 4 046 811 reads with an average length of 211.6 ± 111 bp (SD). From this, approximately 87% of the data had a mean Phred ≥20. After quality control, 2 068 216 reads (51%) were kept, and after random subsampling we retained 1 129 660 reads with an average length of 254.9 ± 56 bp (SD). After clustering at 97% sequence similarity, 10 035 OTUs were generated. From this dataset, we removed 3148 putative chimeras and 1249 singletons. The asymptotic rarefaction curves (Fig.1a) and Good's coverage (Fig.1b) suggest that the deep sequencing allowed for a very high OTU coverage and that likely all fungi present in the samples were sequenced. The final dataset included 343 ECM basidiomycete OTUs (110 665 reads).

**Fig 2:**
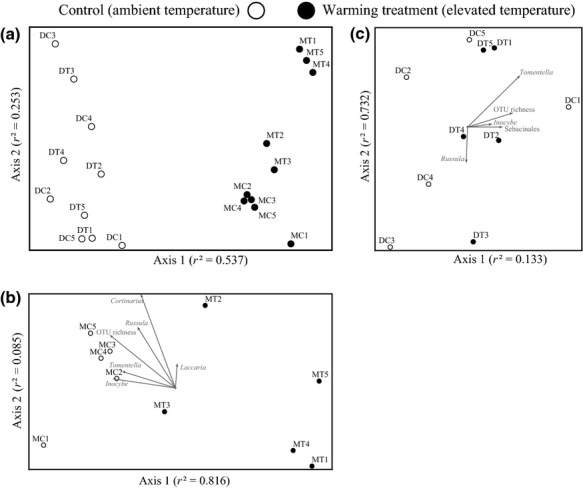
(a) NMDS analysis of the dry and moist tussock tundra with control and treatment sites. (b) NMDS analysis of the ECM fungal communities of the moist tussock tundra replicates. (c) NMDS analysis of the ECM fungal communities of the dry tundra replicates. DC, dry control; DT, dry warming treatment; MC, moist tussock control; MT, moist tussock warming treatment.

We detected 20 ECM basidiomycete genera (Table1). Four of these dominated the communities, accounting for approximately 82% of all OTU richness: *Tomentella* (106 OTUs, 31%), *Cortinarius* (77, 22%), *Inocybe* (63, 18%), and *Russula* (34, 10%). OTU richness in the control plots was not significantly different (*P *=* *0.296, *t*_8_ = 1.119) between the dry and moist tundra types, although the plot-based richness values were somewhat higher in the dry than in the moist tussock tundra (Fig.1c). The NMDS analysis of the full dataset indicated that species assemblages in the dry and moist tundra types are highly dissimilar (Fig.2a). Therefore, we analyzed the results for the two types of tundra separately to focus on the effect of warming on ECM community composition.

**Table 1:** Number of mean OTUs per plot in the control and warming treatment plots in the dry and moist tussock tundra. Significance of treatment effects were determined by comparing the control and treatment plots using Students *t*-test

	Moist tussock tundra			Dry heath tundra		
	Control	Treatment	*P*	Control	Treatment	*P*
*Tomentella*	14.6 ± 6.23	3.8 ± 7.40	0.04*	18.4 ± 12.74	20.8 ± 6.30	0.75
*Cortinarius*	16.6 ± 7.95	7.6 ± 10.33	0.16	8.2 ± 3.49	7.8 ± 7.86	0.92
*Inocybe*	8.2 ± 3.12	1.4 ± 1.14	0.05*	7.4 ± 5.23	5.2 ± 6.76	0.16
*Russula*	6.4 ± 4.16	1.6 ± 2.07	0.002*	3.4 ± 2.88	8.8 ± 7.29	0.62
*Sistotrema*	3 ± 4.12	0.2 ± 0.45	0.17	0.6 ± 0.55	0.6 ± 0.89	1.0
*Tremellodendron*	2.4 ± 2.51	0.2 ± 0.45	0.09	1.4 ± 2.19	0.4 ± 0.55	0.35
*Hebeloma*	2 ± 0	1.2 ± 0.45	0.004*	0.4 ± 0.89	1.4 ± 1.95	0.33
*Leccinum*	2.8 ± 1.64	0.4 ± 0.89	0.021*	0.4 ± 0.55	0.6 ± 0.89	0.68
*Laccaria*	1.4 ± 0.89	1.4 ± 0.55	1.0	0 ± 0	0.8 ± 1.30	0.21
*Clavulina*	0.4 ± 0.55	0.6 ± 0.89	0.68	0.8 ± 0.84	0.8 ± 0.84	1.0
*Alnicola*	0.8 ± 0.45	1 ± 1	0.69	–	–	–
*Pseudotomentella*	–	–	–	0.6 ± 0.89	0.8 ± 0.45	0.67
*Sebacina*	–	0.2 ± 0.45	0.35	0.2 ± 0.45	–	0.35
*Tulasnella*	–	–	–	1.2 ± 1.10	0.8 ± 1.10	0.14
*Clavicorona*	–	–	–	0.2 ± 0.45	0.4 ± 0.55	0.55
*Boletus*	–	–	–	0.6 ± 0.55	0.6 ± 0.55	1.0
*Ceratobasidium*	–	–	–	0.6 ± 0.55	0.2 ± 0.45	0.24
*Lactarius*	0.2 ± 0.45	–	0.35	–	–	–
*Piloderma*	–	–	–	0.2 ± 0.45	–	0.35
*Tomentellopsis*	–	–	–	0	0.4 ± 0.55	0.14
Total community	59 ± 21	20 ± 18	0.013*	45 ± 20	50 ± 19.28	0.66

* Significant treatment effect (*α *= 0.05).

### Moist tussock tundra

The total ECM OTU richness in the warmed plots was approximately half of that in the control plots, 71 and 138, respectively. Similarly, OTU richness per plot was significantly greater in the control, 59 ± 21 (mean ± SD) OTUs per plot, than in the treatment (20 ± 18) (*t*_8_ = 3.19, *P *=* *0.013) (Table1). NMDS analyses of the presence–absence matrix resulted in a 2-dimensional solution with a final stress of 0.0395 and a final instability <0.00001. The two axes explained the majority of variability in the sampled fungal communities (axis 1: *r*^*2*^ = 0.816; axis 2: *r*^*2*^ = 0.085; total *r*^*2*^ = 0.901; orthogonality = 88.5%). The NMDS ordination plot was orthogonally rotated by the treatment to visualize correlations between warming and fungal community composition in general, and the taxonomic groups in particular. The MRPP analysis suggested a significant correlation between community composition and the warming treatment (*A *=* *0.12345835, *P *=* *0.0066) that was visually depicted on the NMDS ordination plot (Fig.2b). The NMDS and MRPP results obtained from the square-root abundance were very similar to the presence-absence based results (Figure S1a).

**Fig 3:**
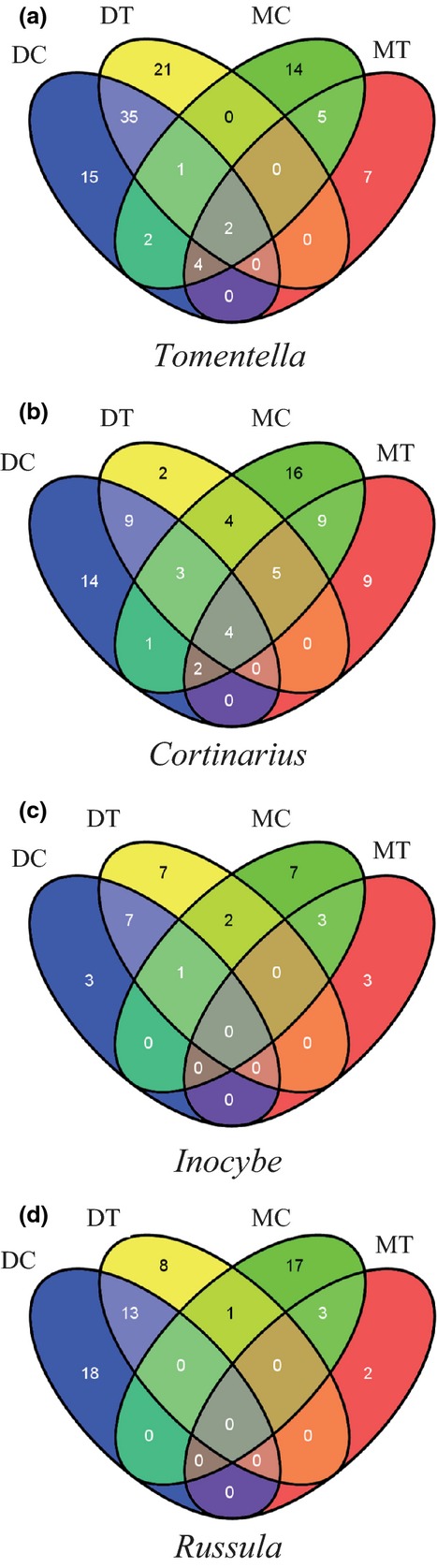
Venn diagrams of the four most diverse genera. DC, dry control; DT, dry warming treatment; MC, moist tussock control; MT, moist tussock warming treatment.

C*ortinarius* was the genus with the highest richness, followed by *Tomentella*, *Russula*, and *Inocybe* (Table1). Several groups that were present in dry tundra were not detected in moist tussock tundra: *Boletus*, *Ceratobasidium*, *Piloderma*, *Pseudotomentella*, *Tomentellopsis,* and *Tulasnella*. On the other hand, *Alnicola* and *Lactarius* were only found in the moist tussock tundra and were generally rare there as well (Table1). In the four dominant genera, only 26% of the OTUs were present in both the control and the treatment plots, and most of the OTUs were only found in the control plots (Fig.3). OTU richness values were significantly lower in the treatment plots, except in *Cortinarius* where the decrease in per-plot OTU richness between control and treatment was not significant.

OTU richness values in most genera were nega-tively correlated with the warming, with *Hebeloma* (*r *=* *−0.909), *Inocybe* (*r *=* *−0.751), and *Tomentella* (*r *=* *−0.691) as well as total OTU richness (*r *=* *−0.768) showing the strongest correlation. On the other hand, *Laccaria* and *Alnicola* did not seem to be influenced by the treatment (*r *=* *0.126 and *r *=* *0.108, respectively), perhaps due, in part, to their rarity. The indicator species analysis revealed 14 OTUs significantly associated with the control plots, while none of the OTUs were found to be indicators of the treatment plots (Table2).

**Table 2:** Indicator species analysis of OTUs with significant correlation (*α *= 0.05) with the site, their taxonomic affinity and similarity with referenced species hypothesis (SH) and/or known sequences from UNITE database or GenBank

OTU	Correlated site	Kõljalg *et al*. (2013) and UNITE classification	Similarity (%)
1281	DC	SH112690.05FU – *Tomentella coerulea* (UDB016493)	97.9
3369	MC	SH115895.05FU – *Leccinum holopus* (UDB001378)	99.6
484	MC	SH115895.05FU – *Tomentella fuscocinerea* (UDB016484)	99.6
3351	MC	SH108145.05FU – *Tomentella lateritia* (UDB016439)	97.8
181	MC	SH112435.05FU – *Tomentella coerula* (UDB018451)	98.1
4645	MC	SH108158.05FU – *Tomentella* sp. (UDB017832)	98.9
6618	MC	SH108158.05FU – *Hebeloma collariatum* (UDB17969)	96.2
1120	MC	SH102330.05FU – *Russula renidens* (UDB015975)	100
4313	MC	SH102330.05FU – *Tomentella fuscocinerea* (UDB016188)	95.9
1124	MC	SH102330.05FU – *Tomentella fuscocinerea* (UDB016188)	99.6
1625	MC	SH166458.05FU – *Cortinarius croceus* (UDB011339)	99.7
801	MC	SH111588.05FU – *Inocybe nitidiuscula* (HQ604382)	96.6
3413	MC	SH111588.05FU – *Inocybe nitidiuscula* (HQ604382)	95.9
5841	MC	SH099601.05FU – *Inocybe leiocephala* (AM882793)	96.7
219	MC	SH099601.05FU – *Inocybe leiocephala* (AM882793)	99

**Fig 4:**
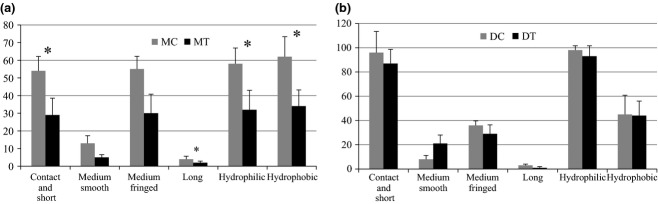
(a) Number of OTUs per plot per extramatrical mycelium characteristics in the moist tussock control vs. moist tussock warming treatment plots with standard deviation of the five replicates. (b) Number of unique OTUs per extramatrical mycelium characteristics in the dry control plots vs. dry warming treatment plots with standard deviation of the five replicates. *Significant treatment effect (α = 0.05).

Two EMM types dominated the community, the medium-distance fringe and the contact/short-distance type, in both the control and the treatment plots (Fig.4a). There was a significant decrease in the number of OTUs of most of the EMM types in the treatment plots. However, the effects in the medium-distance fringe types were not statistically significant.

### Dry tundra

OTU richness in the control and treatment plots did not differ significantly (*t*_8_ = 0.46, *P *=* *0.66) (Table1) with 45 ± 20 and 50 ± 19 OTUs per plot, respectively. T*omentella* was the most OTU-rich genus (having nearly double the amount of total number of OTUs, than the second most diverse taxonomic group), followed by *Cortinarius*, *Russula,* and *Inocybe* (Table1). Approximately 40% of the OTUs were present in both the control and the treatment plots (Fig.3). In the dominant genera, the relative frequency of OTUs present in both the control and treatment plots was relatively high (compared with the values obtained for the moist tundra), varying from 33% in *Russula* to 48% in *Tomentella* (Fig.3).

The MRPP analysis suggested no significant correlation between community composition and treatment (*A *=* *−0.00147, *P *=* *0.4288), which was confirmed by the NMDS analysis (Fig.2c). Again, the NMDS and MRPP results obtained from the square-root abundance matrix were very similar to the presence-absence based results (Supporting information, Fig. S1b). However, Pearson's correlation values suggested that OTU richness in *Tomentella* (*r *=* *0.789), Sebacinales (*Sebacina* and *Tremellodendron*) (*r *=* *0.640), and *Inocybe* (*r *=* *0.535), as well as the total OTU richness (*r *=* *0.730) were positively correlated with the treatment. Even though the remaining groups did not show strong correlation with warming, the genera *Russula* and *Laccaria* exhibited an interesting pattern. Although the mean richness of *Russula* did not differ significantly (*t*_8_ = 1.5397, *P *=* *0.1622) in the control and treatment plots (3 ± 3 and 9 ± 7 OTUs per plot, respectively), the total number of *Russula* OTUs with EMM medium-distance smooth type in the treatment plots was considerably higher than in the control plots (17 and 7, respectively). Also, *Laccaria* OTUs were only found in the treatment plots. Species from this genus have been argued to (i) possess an EMM of the medium-distance smooth exploration type with hydrophilic hyphae (Unestam x0026; Sun, 1995; Agerer, 2001) and (ii) to be nitrophilic with positive response to disturbance (Dickie x0026; Moyersoen, 2008). The indicator species analysis (Table2) revealed that OTU 1281, identified as *Tomentella atramentaria* (SH112690.05FU), was negatively correlated with warming.

We found that the majority of the OTUs were of the contact and short distance EMM type with hydrophilic hyphae, in both the control and the treatment plots. There was a nonsignificant decrease in the number of OTUs of most of the EMM types in the treatment plots. However, the medium-distance smooth type showed an opposite pattern, having an increment in the number of OTUs in the treatment plots and this difference was marginally significant (*t*_8_ = 2.28, *P = *0.0567).

## Discussion

### Diversity

We found 343 OTUs of ECM basidiomycetes in the sampled moist tussock and dry tundra in Alaskan Arctic. These OTUs were spread across 20 genera. This richness is the highest ever reported for arctic ECM fungi. Previous studies on belowground diversity of arctic ECM fungi that used similar methods, reported between 73 and 202 OTUs of ca. 12 genera (Bjorbækmo *et al*., 2010; Geml *et al*., 2012; Timling x0026; Taylor, 2012; Timling *et al*., 2012, 2014). Moreover, several genera remain undersampled in our dataset (e.g., *Lactarius*, *Amanita*), possibly due to their small genet size and relative rarity at the landscape scale. Because observed fruitbodies of several *Amanita* and *Lactarius* species near the sampled plots, in identical vegetation types, it is likely that the real diversity of ECM fungi in the sampled region is even higher than our estimates.

In general, the dominant taxonomic groups that we uncovered, *Tomentella*, *Cortinarius*, *Inocybe*, and *Russula*, are agreement with the findings of previous studies that used molecular techniques to study belowground diversity in arctic tundra communities (Bjorbækmo *et al*., 2010; Geml *et al*., 2012; Timling x0026; Taylor, 2012; Timling *et al*., 2012). On the other hand, the dry and moist tundra types were dominated by distinct taxonomic groups, namely *Tomentella* and *Cortinarius*, respectively (Table1). Such a difference was also apparent in the EMM types that were found more prevalent in the different tundra types. While there seems to be a codominance of two EMM types (medium-distance and contact/short distance) in the moist tussock tundra with equal richness of OTUs with hydrophobic and hydrophilic hyphae; in the dry tundra, only the contact/short-distance EMM type with hydrophobic hyphae were dominant (Fig.4b).

### Warming-induced changes in the moist tussock tundra

Our results show a clear decrease in ECM fungal richness in response to warming in the moist tussock tundra, which is in clear contradiction with the single previous study addressing the effects of long-term warming on ECM fungal diversity in the Arctic (Deslippe *et al*., 2011). Deslippe *et al*. (2011) reported a warming-induced increase in diversity of ECM fungi associated with *Betula nana*. The contradiction might be due to methodological differences and the sampling depth of the ECM communities. While our data are derived from soil associated with the whole plant community, and comprised 110 684 sequences that were clustered into 343 OTUs, the observations of Deslippe *et al*. (2011) were based on 1060 nonclustered sequences (ca. 70 OTUs) derived from cloning of root tips of a single ECM host, *B. nana*. The steep OTU rarefaction curves generated by Deslippe *et al*. (2011) suggest that only a fraction of all ECM taxa at the sites were sequenced. Therefore, their sampling intensity likely was inadequate to obtain near-complete coverage to capture changes in richness. Due to our deep sequencing efforts, our rarefaction curves indicate that the vast majority of fungal taxa in the sampling sites have been sequenced that, in turn, provides a more solid base for among-site comparisons.

*Cortinarius* was the only dominant genus with nonsignificant decrease in per-plot OTU richness. *Cortinarius* also stands out by having most OTUs present in both control and treatment plots, a pattern that is distinct from the other three dominant genera (Fig.3). This suggests that most *Cortinarius* spp in the moist tundra may be resilient and/or adapted to the conditions induced by warming. In light of the EMM characteristics, it is interesting to note that, contrary to the other three dominant genera, *Cortinarius* has an EMM with medium-distance fringed exploration type and hydrophobic rhizomorphs (Agerer, 2001, 2006). Taking into account that prior evidence point to a lack of effect of warming on ECM colonization ratios and that ECM fungal biomass increases with warming (Clemmensen *et al*., 2006), it seems reasonable to hypothesize that *Cortinarius* spp. might have an advantage over other ECM taxa under long-term summer temperature increase.

Lilleskov *et al*. (2011) suggested that fungi with medium-distance fringed exploration type and hydrophobic rhizomorphs, such as *Cortinarius*, are likely to have hydrolytic capabilities that would facilitate acquisition and translocation of insoluble proteins. It has been postulated for an extended period that most extracellular enzyme secretion is likely to be confined to the hyphae tip close to the apex, where the wall pore size is large enough to be permeable to enzymes of relatively large molecular weight (e.g., Chang x0026; Trevithick, 1974; Unestam x0026; Sun, 1995; Lindahl *et al*., 2005). Indeed, the functional compartmentalization in gene expression between root tips and foraging mycelium (Wright *et al*., 2005), coupled with the very low activity of enzymes correlated with ECM fungi measured on ECM root tips compared to levels of activity measured in bulk soils (Talbot *et al*., 2013) support the hypothesis that the EMM have a crucial role in nutrient acquisition. Recently, Bödeker and colleagues (2014) provided compelling evidence supporting the hypothesis that at least, some *Cortinarius* spp. are directly involved in soil organic matter degradation through extracellular enzymatic activity. This capability coupled with a diffuse mycelium may be advantageous for *Cortinarius* spp. to colonize recalcitrant and unevenly distributed nutrient soil pools, nutrients uptake, and to translocate them from the soil to the host roots while promoting root connectivity (Hobbie x0026; Agerer, 2010; Lilleskov *et al*., 2011). Such functional roles can facilitate plant mineral nutrition resulting in higher plant and leaf N, as well as greater growth and greater leaf photosynthesis as observed in the warmed plots by Welker *et al*. (2005) and by Pattison x0026; Welker (2014). In exchange the host plant might increase carbohydrates allocation to the root system and EMM as a tradeoff, justifying the high energetic investment by the ECM fungi. Such mechanisms of soil C accumulation derived from roots and root-associated fungi have been shown to contribute 50–70% of stored C in ECM-dominated boreal forests (Clemmensen *et al*., 2013).

The genus *Tomentella* shows a similar pattern of OTU distribution as *Cortinarius* – high relative frequency (31% and 38%, respectively) of OTUs that are present in both control and treatment plots (Fig.3). This pattern is distinct from that observed in *Russula* and *Inocybe*, both of which have a low relative frequency (13% and 19%, respectively) of OTUs present in both the control and the treatment plots. A couple of hypotheses can be raised to interpret this trend. First, the EMM characteristics of *Tomentella* spp. are still largely unknown and there is some evidence that EMM morphology varies considerably within this diverse genus. There are species with EMM with variable hydrophobicity and exploration types, such as contact, short distance and medium-distance (Agerer, 2001, 2006; Hobbie x0026; Agerer, 2010). Therefore, it is possible that the EMM characteristics of the OTUs affiliated with *Tomentella* are more varied than initially assumed and distinct from the OTUs affiliated with *Russula* and *Inocybe*.

OTU richness of taxa having an EMM with the long-distance type was very low when compared to the medium-distance fringe type (Fig.4a). In the moist tussock tundra, all OTUs with EMM of the long-distance type, that has been hypothesized to play an important role in nutrient translocation and new root-colonization (Hobbie x0026; Agerer, 2010; Weigt *et al*., 2011), were affiliated with *Leccinum* spp. that is specific to *Betula nana* in the Arctic. It has been hypothesized that *Leccinum* species have proteolyctic capabilities (Nygren *et al*., 2008; Eaton x0026; Ayres, 2002). Therefore, it is surprising that the OTU richness with this EMM type decreased significantly in the treatment plots, since we expected a similar pattern as in the species with medium-distance fringe exploration type (for in-depth discussion for potential similar patterns between these two groups consult Hobbie x0026; Agerer (2010)). However, the significance of this decrease might be influenced by the overall low diversity of taxa with long-distance EMM type in the arctic tundra. It is possible that these changes in diversity might not relate to actual differences in overall ecological function, since two of 4 OTUs that represented this EMM type are present in the treatment plots.

It is important to understand that we do not claim that temperature increase affects the fungi directly. Instead, we argue that the warming induces changes in the entire biotic community and, via the numerous intimate interactions fungi have with other living organisms in these plots, changes are apparent in the ECM fungal community as well, regardless if they are directly or indirectly caused by warming. Because OTCs minimize unwanted ecological effects while effectively elevating temperature (Marion *et al*., 1997), it is reasonable to assume that most of the changes in the fungal community composition are induced by increased air and surface temperature. However, it is unclear if shifts in ECM fungal communities are due to direct effects of temperature on fungal metabolism or caused indirectly, e.g., via changes in plant-fungus interactions, rather than by the direct effect of temperature on fungal metabolism (or, likely, are combinations of the two). On the other hand, it is conceivable that other factors (e.g., leaf temperature increase on sunny days) may or may not have some indirect influence on the ECM fungi associated with these plants. Due to the lack of information and relevant empirical data, it is difficult to even speculate about possible mechanisms and disentangling such causal relationships are beyond the scope of this paper.

### Resilience in the dry tundra

Our results do not show significant differences in either ECM fungal community composition or richness in the dry tundra. Former vegetation studies on dry tundra community also indicated no effect of warming on plant species richness, but there were significant increases in shrub canopy height and cover, mainly due to increase in evergreen shrubs (Mercado-Díaz, 2011). These changes in plant community only occurred more than 8 years after initiating the experiment (Walker *et al*., 1999; Wahren *et al*., 2005; Mercado-Díaz, 2011) reflecting a less pronounced effect of the warming treatment on the dry tundra than what is reported for the moist tussock tundra (see above). Contrary to the patterns observed in the moist tundra, the changes in the dry tundra vegetation were mainly in non-ECM plants. Therefore, the absence of significant changes in the ECM fungal community of the dry tundra is in agreement with the lack of compositional changes in the ECM plant hosts. However, since the significant shifts in the vegetation are in non-ECM hosts (Mercado-Díaz, 2011), we hypothesize that other root-associated fungi, particularly ericoid mycorrhizal fungi, may exhibit stronger response to warming.

Although the NMDS analysis indicated no significant changes in the ECM fungal community composition, we found an interesting pattern in two taxonomic groups at the functional level, i.e., in the EMM types. For example, in the control plots, the genus *Russula* was represented by two EMM types: the contact with hydrophilic hyphae and the medium-distance smooth type with varying hydrophobicity. On the other hand, only OTUs with medium-distance type with mainly hydrophilic hyphae were detected in the warmed plots. Similarly, the genus *Laccaria* that has EMM of the medium-distance smooth type with hydrophilic hyphae, was only found in the treatment plots. Even though this pattern might result from chance alone, it can also be indicative of slow and/or minute shifts in the community.

When analyzing the EMM of the OTUs that compose the dry tundra community, it is interesting to note that the contact and short-distance exploration types with hydrophilic hyphae were represented by more than twice as many OTUs as the medium-distance exploration type with hydrophobic rhizomorphs. The majority of ECM fungi with contact and short-distance exploration types with hydrophilic hyphae have been hypothesized to explore the pools of labile nutrients in the soil, since most of them showed reduced proteolytic capabilities in laboratory experiments (Lilleskov *et al*., 2002; Nygren *et al*., 2007). Recently, Hobbie *et al*. (2013), using radiocarbon data, provided evidence from natural ecosystems that also support this hypothesis. Lilleskov *et al*. (2011) hypothesized that taxa with this EMM type are predominant in conditions of low below ground C allocation, because they probably have a low C cost to the host. This is agreement with previous findings that suggest that the dry tundra has far less productivity than the moist tundra (Gough *et al*., 2007). Unfortunately, the scarcity of knowledge on the nutrient acquisition strategies of most ECM fungi prevent us from further speculations about possible warming-induced functional changes in the community and their effects on nutrient cycling.

### High contrast in ECM communities' responses to warming at the low Arctic

This article is the first to study the effects of long-term experimental warming in the ECM fungal community at a habitat-scale of the low Arctic tundra using high-throughput sequencing techniques. We provide evidence that (i) long-term summer temperature increases have contrasting effects on various ECM fungal genera; and (ii) these effects are habitat-dependent. These patterns might be indicative of ecological strategies of various ECM fungi as well as of differences in patterns of C storage and N cycling in these two tundra types (Welker *et al*., 2000; Schimel *et al*., 2004; Welker *et al*., 2005; Pattison x0026; Welker, 2014). The nonsignificant changes in richness and composition of the ECM fungal community in the dry tundra (this study) coupled with evidence of nonsignificant changes in the ECM fungal biomass in a subarctic heath tundra, near Abisko, Sweden, (Clemmensen *et al*., 2006) might indicate that the biogeochemical processes in the dry tundra remain largely unaltered with moderate warming. Also, environmental changes other than air and surface temperature, might play a more important role in this tundra type, e.g., changes in snow depth have been reported to influence microbial activity and N cycle (Schimel *et al*., 2004), which in turn might also affect the ECM fungal community. In the moist tundra, the observed changes in the ECM fungal community (this study) coupled with the increase in ECM fungal biomass (Clemmensen *et al*., 2006) suggest increased capability of N mobilization, which might be derived from recalcitrant soil pools. This may favor enhanced C allocation from the plants to the ECM fungi and the rhizosphere, and, therefore, enhanced C storage in the soil biotic community. Even though these processes might surpass soil organic matter decomposition and microbial respiration rates, and therefore, contribute to positive feedbacks in climate change, this still remains an open question that needs further investigations.

## Supporting Information

Figure S1 (a) NMDS analysis of the ECM fungal communities of the moist tussock tundra plots with final stress 0.0523, final instability <0.00001, total *r*^*2*^ = 0.876 and orthogonality = 96.5%. MRPP *A *=* *0.0927 and *P *=* *0.0051. (b) NMDS analysis of the ECM fungal communities of the dry tundra replicates with final stress 0.1266 and final instability <0.00001. MRPP *A *=* *0.0163 and *P *=* *0.1750. DC, dry control; DT, dry warming treatment; MC, moist tussock control; MT, moist tussock warming treatment.

## References

[b1] Abarenkov K, Nilsson RH, Larsson K-H (2010). The UNITE database for molecular identification of fungi – recent updates and future perspectives. New Phytologist.

[b2] Agerer R (2001). Exploration types of ectomycorrhizae. A proposal to classify ectomycorrhizal mycelial systems according to their patterns of differentiation and putative ecological importance. Mycological Progress.

[b3] Agerer R (2006). Fungal relationships and structural identity of their ectomycorrhizae. Mycological Progress.

[b4] Amend AS, Seifert KA, Bruns TD (2010). Quantifying microbial communities with 454 pyrosequencing: does read abundance count?. Molecular Ecology.

[b5] Anderson IC, Cairney JWG (2007). Ectomycorrhizal fungi: exploring the mycelial frontier. FEMS Microbiology Reviews.

[b6] Anderson-Smith M (2013). Remotely-sensed spectral data linked to increasing shrub abundance and greater growing season carbon uptake in Alaska Arctic Tundra.

[b7] Anisimov OA, Vaughan DG, Callaghan TV, Parry ML, Canziani OF, Palutikof JP, dervan LindenPJ, Hanson CE (2007). Polar regions (Arctic and Antarctic). Climate Change 2007: Impacts, Adaptation and Vulnerability.

[b8] Arft AM, Walker MD, Gurevitch J (1999). Responses of tundra plants to experimental warming: meta-analysis of the international tundra experiment. Ecological Monographs.

[b9] Arrigo KR, van Dijken GL (2011). Secular trends in Arctic Ocean net primary production. Journal of Geophysical Research: Oceans.

[b10] Bjorbækmo MFM, Carlsen T, Brysting A (2010). High diversity of root associated fungi in both alpine and arctic *Dryas octopetala*. BMC Plant Biology.

[b11] Blaalid R, Kumar S, Nilsson RH, Abarenkov K, Kirk PM, Kauserud H (2013). ITS1 versus ITS2 as DNA metabarcodes for fungi. Molecular Ecology Resources.

[b12] Bödeker ITM, Clemmensen KE, de Boer W, Martin F, Olson A, Lindahl BD (2014). Ectomycorrhizal *Cortinarius* species participate in enzymatic oxidation of humus in northern forest ecosystems. New Phytologist.

[b13] Bokhorst S, Huiskes A, Aerts R (2013). Variable temperature effects of open top chambers at polar and alpine sites explained by irradiance and snow depth. Global Change Biology.

[b14] Borner AP, Kielland K, Walker MD (2008). Effects of simulated climate change on plant phenology and nitrogen mineralization in Alaskan Arctic Tundra effects of simulated climate change on plant phenology and nitrogen mineralization in Alaskan Arctic Tundra. Arctic, Antarctic, and Alpine Research.

[b15] Boyle H, Zimdars B, Renker C, Buscot F (2006). A molecular phylogeny of *Hebeloma* species from Europe. Mycological Research.

[b16] Brown SP, Callaham MA, Oliver AK, Jumpponen A (2013). Deep Ion Torrent sequencing identifies soil fungal community shifts after prescribed fires in a southeastern US forest ecosystem. FEMS Microbiology Ecology.

[b17] Cahoon SMP, Sullivan PF, Shaver GR, Welker JM, Post E, Holyoak M (2012). Interactions among shrub cover and the soil microclimate may determine future Arctic carbon budgets. Ecology Letters.

[b18] Cairney JWG (2012). Extramatrical mycelia of ectomycorrhizal fungi as moderators of carbon dynamics in forest soil. Soil Biology and Biochemistry.

[b1000] Cairney JWG, Burke RM (1996). Physiological heterogeneity within fungal mycelia: an important concept for a functional understanding of the ectomycorrhizal symbiosis. New Phytologist.

[b19] Campbell BJ, Polson SW, Hanson TE, Mack MC, Schuur EAG (2010). The effect of nutrient deposition on bacterial communities in Arctic tundra soil. Environmental Microbiology.

[b20] Chang PL, Trevithick JR (1974). How important is secretion of exoenzymes through apical cell walls of fungi?. Archives of Microbiology.

[b21] Chapin FS, Shaver GR (1985). Individualistic growth response of tundra plant species to environmental manipulations in the field. Ecology.

[b22] Chapin FS, Sturm M, Serreze MC (2005). Role of land-surface changes in Arctic summer warming. Science.

[b23] Clemmensen KE, Michelsen A, Jonasson S, Shaver GR (2006). Increased ectomycorrhizal fungal abundance after long-term fertilization and warming of two arctic tundra ecosystems. New Phytologist.

[b24] Clemmensen KE, Bahr A, Ovaskainen O (2013). Roots and associated fungi drive long-term carbon sequestration in boreal forest. Science.

[b25] Colpaert JV, van Assche JA, Luijtens K (1992). The growth of the extramatrical mycelium of ectomycorrhizal fungi and the growth response of *Pinus sylvestris* L. New Phytologist.

[b26] Comiso JC, Hall DK (2014). Climate trends in the Arctic as observed from space. Wiley Interdisciplinary Reviews: Climate Change.

[b27] Cornelissen JHC, Callaghan TV, Alatalo JM (2001). Global change and arctic ecosystems: is lichen decline a function of increases in vascular plant biomass?. Journal of Ecology.

[b28] Courty P-E, Franc A, Pierrat J-C, Garbaye J (2008). Temporal changes in the ectomycorrhizal community in two soil horizons of a temperate oak forest. Applied and Environmental Microbiology.

[b29] Deslippe JR, Hartmann M, Mohn WW, Simard SW (2011). Long-term experimental manipulation of climate alters the ectomycorrhizal community of *Betula nana* in Arctic tundra. Global Change Biology.

[b30] Deslippe JR, Hartmann M, Simard SW, Mohn WW (2012). Long-term warming alters the composition of Arctic soil microbial communities. FEMS Microbiology Ecology.

[b31] Dickie IA, Moyersoen B (2008). Towards a global view of ectomycorrhizal ecology. New Phytologist.

[b32] Dickie IA, Reich PB (2005). Ectomycorrhizal fungal communities at forest edges. Journal of Ecology.

[b33] Dickie IA, Schnitzer SA, Reich PB, Hobbie SE (2005). Spatially disjunct effects of co-occurring competition and facilitation. Ecology Letters.

[b34] Downing AS, van Nes EH, Mooij WM, Scheffer M (2012). The resilience and resistance of an ecosystem to a collapse of diversity. PLoS ONE.

[b35] Dufrêne M, Legendre P (1997). Species assemblages and indicator species: the need for a flexible assymetrical approach. Ecological Monographs.

[b36] Eaton GK, Ayres MP (2002). Plasticity and constraint in growth and protein mineralization of ectomycorrhizal fungi under simulated nitrogen deposition. Mycologia.

[b37] Edgar RC (2010). Search and clustering orders of magnitude faster than BLAST. Bioinformatics.

[b38] Elmendorf SC, Gregory HR, Henry RD (2012). Global assessment of experimental climate warming on tundra vegetation: heterogeneity over space and time. Ecology Letters.

[b39] Finlay RD (2008). Ecological aspects of mycorrhizal symbiosis: with special emphasis on the functional diversity of interactions involving the extraradical mycelium. Journal of Experimental Botany.

[b40] Gardes M, Dahlberg A (1996). Mycorrhizal diversity in arctic and alpine tundra: an open question. New Phytologist.

[b41] Geml J, Laursen GA, Taylor DL (2008). Molecular diversity assessment of arctic and boreal *Agaricus* taxa. Mycologia.

[b42] Geml J, Laursen GA, Timling I (2009). Molecular phylogenetic biodiversity assessment of arctic and boreal *Lactarius* Pers. (Russulales; Basidiomycota) in Alaska, based on soil and sporocarp DNA. Molecular Ecology.

[b43] Geml J, Timling I, Robinson CH (2012). An arctic community of symbiotic fungi assembled by long-distance dispersers: phylogenetic diversity of ectomycorrhizal basidiomycetes in Svalbard based on soil and sporocarp DNA. Journal of Biogeography.

[b44] Geml J, Gravendeel B, Neilen M, Lammers Y, Raes N, Semenova TA, Noordeloos ME (2014a). DNA metabarcoding reveals high fungal diversity and pH-correlated habitat partitioning in protected coastal *Salix repens* communities in the Netherlands. PLoS ONE.

[b45] Geml J, Pastor N, Fernandez L (2014b). Large-scale fungal diversity assessment in the Andean Yungas forests reveals strong community turnover among forest types along an altitudinal gradient. Molecular Ecology.

[b46] Gihring TM, Green SJ, Schadt CW (2012). Massively parallel rRNA gene sequencing exacerbates the potential for biased community diversity comparisons due to variable library sizes. Environmental Microbiology.

[b47] Gough L, Ramsey EA, Johnson DR (2007). Plant-herbivore interactions in Alaskan arctic tundra change with soil nutrient availability. Oikos.

[b48] Hansen J, Ruedy R, Sato M, Lo K (2010). Global surface temperature change. Reviews of Geophysics.

[b49] Hartley IP, Garnett MH, Sommerkorn M (2012). A potential loss of carbon associated with greater plant growth in the European Arctic. Nature Climate Change.

[b50] Higgins KL, Arnold AE, Miadlikowska J, Sarvate SD, Lutzoni F (2007). Phylogenetic relationships, host affinity, and geographic structure of boreal and arctic endophytes from three major plant lineages. Molecular Phylogenetics and Evolution.

[b51] Hobbie EA, Agerer R (2010). Nitrogen isotopes in ectomycorrhizal sporocarps correspond to belowground exploration types. Plant and Soil.

[b52] Hobbie JE, Hobbie EA (2006). ^15^N in symbiotic fungi and plants estimates nitrogen and carbon flux rates in Arctic tundra. Ecology.

[b53] Hobbie JE, Hobbie EA, Drossman H, Conte M, Weber JC, Shamhart J, Weinrobe M (2009). Mycorrhizal fungi supply nitrogen to host plants in Arctic tundra and boreal forests: ^15^N is the key signal. Canadian Journal of Microbiology.

[b54] Hobbie EA, van Diepen LTA, Lilleskov EA, Ouimette AP, Finzi AC, Hofmockel KS (2013). Fungal functioning in a pine forest: evidence from a (15) N-labeled global change experiment. New Phytologist.

[b55] Ihrmark K, Bödeker ITM, Cruz-Martinez K (2012). New primers to amplify the fungal ITS2 region–evaluation by 454-sequencing of artificial and natural communities. FEMS Microbiology Ecology.

[b56] Jägerbrand AK, Alatalo JM, Chrimes D, Molau U (2009). Plant community responses to 5 years of simulated climate change in meadow and heath ecosystems at a subarctic-alpine site. Oecologia.

[b57] Johnson D, Martin F, Cairney JWG, Anderson IC (2012). The importance of individuals: intraspecific diversity of mycorrhizal plants and fungi in ecosystems. New Phytologist.

[b58] Kade A, Walker DA, Raynolds MK (2005). Plant communities and soils in cryoturbated tundra along a bioclimate gradient in the Low Arctic, Alaska. Phytocoenologia.

[b1001] Kauserud H, Kumar S, Brysting AK, Nordén J, Carlsen T (2012). High consistency between replicate 454 pyrosequencing analyses of ectomycorrhizal plant root samples. Mycorrhiza.

[b59] Kharuk VI, Ranson KJ, Sergey IT, Oskorbin PA, Dvinskaya ML, Ovchinnikov DV (2013). Tree-line structure and dynamics at the northern limit of the Larch forest: Anabar Plateau, Siberia, Russia. Arctic, Antarctic, and Alpine Research.

[b60] Koide RT, Fernandez C, Malcolm G (2014). Determining place and process: functional traits of ectomycorrhizal fungi that affect both community structure and ecosystem function. New Phytologist.

[b61] Kõljalg U, Nilsson RH, Abarenkov K (2013). Towards a unified paradigm for sequence-based identification of Fungi. Molecular Ecology.

[b62] Lilleskov EA, Fahey TJ, Horton TR, Lovett GM (2002). Belowground ectomycorrhizal community change over a nitrogen deposition gradient in Alaska. Ecology.

[b63] Lilleskov EA, Hobbie EA, Horton TR (2011). Conservation of ectomycorrhizal fungi: exploring the linkages between functional and taxonomic responses to anthropogenic N deposition. Fungal Ecology.

[b64] Lindahl BD, Finlay RD, Cairney JWG, Dighton J, Oudemans P, White J (2005). Enzymatic activities of mycelia in mycorrhizal fungal communities. The Fungal Community, its Organization and Role in the Ecosystem.

[b65] Lindahl BD, Nilsson RH, Tedersoo L (2013). Fungal community analysis by high-throughput sequencing of amplified markers–a user's guide. New Phytologist.

[b1002] Loranty MM, Goetz SJ (2012). Shrub expansion and climate feedbacks in Arctic tundra. Environmental Research Letters.

[b66] Marion GM, Henry GHR, Freckman DW (1997). Open-top designs for manipulating field temperature in high-lattitude ecosystems. Global Change Biology.

[b67] Martin F, Duplessis S, Ditengou F, Lagrange H, Voiblet C, Lapeyrie F (2001). Developmental cross talking in the ectomycorrhizal symbiosis: signals and communication genes. New Phytologist.

[b68] McCune B, Grace JB (2002). Analysis of Ecological Communities.

[b69] Mercado-Díaz J (2011).

[b70] Miller SL, Buyck B (2002). Molecular phylogeny of the genus *Russula* in Europe with a comparison of modern infrageneric classifications. Mycological Research.

[b71] Nilsson RH, Abarenkov K, Larsson K-H, Köljalg U (2011). Molecular identification of fungi: rationale, philosophical concerns, and the UNITE database. The Open Applied Informatics Journal.

[b72] Nygren CMR, Edqvist J, Elfstrand M, Heller G, Taylor AFS (2007). Detection of extracellular protease activity in different species and genera of ectomycorrhizal fungi. Mycorrhiza.

[b73] Nygren C, Eberhardt U, Karlsson M, Parrent JL, Lindahl B, Taylor AFS (2008). Growth on nitrate and occurrence of nitrate reductase-encoding genes in a phylogenetically diverse range of ectomycorrhizal fungi. New Phytologist.

[b74] O'Brien H, Parrent JL, Jackson JA, Moncalvo JM, Vilgalys R (2005). Fungal community analysis by large-scale sequencing of environmental samples. Applied and Environmental Microbiology.

[b75] Oksanen J, Guillaume Blanchet F, Kindt R (2012). http://CRAN.R-project.org/package=vegan.

[b76] Oliveros JC (2007). http://bioinfogp.cnb.csic.es/tools/venny/index.html.

[b77] Pattison RR, Welker JM (2014). Differential ecophysiological response of deciduous shrubs and a graminoid to long-term experimental snow reduction and addition in moist tundra, Nothern Alaska. Oecologia.

[b78] Peay KG, Kennedy PG, Bruns TD (2011). Rethinking ectomycorrhizal succession: are root density and hyphal exploration types drivers of spatial and temporal zonation?. Fungal Ecology.

[b79] Post E, Forchhammer MC, Bret-Harte MS (2009). Ecological dynamics across the Arctic associated with recent climate change. Science.

[b80] Post E, Bhatt US, Bitz CM (2013). Ecological consequences of sea-ice decline. Science.

[b81] Read DJ, Leake JR, Perez-Moreno J (2004). Mycorrhizal fungi as drivers of ecosystem processes in heathland and boreal forest biomes. Canadian Journal of Botany.

[b82] Ryberg M, Larsson E, Molau U (2009). Ectomycorrhizal diversity on *Dryas octopetala* and *Salix reticulata* in an alpine cliff ecosystem. Arctic, Antarctic, and Alpine Research.

[b83] Schaeffer SM, Sharp E, Schimel JP, Welker JM (2013). Soil-plant processes in a high Arctic ecosystem, NW Greenland are altered by long-term warming and higher rainfall. Global Change Biology.

[b84] Schimel JP, Bilbrough C, Welker JM (2004). Increased snow depth affects microbial activity and nitrogen mineralization in two arctic tundra communities. Soil Biology x0026; Biochemistry.

[b85] Schloss PD, Westcott SL, Ryabin T (2009). Introducing mothur: open-source, platform-independent, community-supported software for describing and comparing microbial communities. Applied Environmental Microbiology.

[b86] Schloss PD, Gevers D, Westcott SL (2011). Reducing the effects of PCR amplification and sequencing artifacts on 16S rRNA-based studies. PLoS ONE.

[b87] Selosse M-A, Richard F, He X, Simard SW (2006). Mycorrhizal networks: des liaisons dangereuses?. Trends in Ecology x0026; Evolution.

[b1003] Sharkhuu A, Plante AF, Enkhmandal O, Casper BB, Helliker BR, Boldgiv B, Petraitis PS (2013). Effects of open-top passive warming chambers on soil respiration in the semi-arid steppe to taiga forest transition zone in Northern Mongolia. Biogeochemistry.

[b88] Sharp E, Sullivan P, Steltzer H, Csank A, Welker JM (2013). Complex carbon cycling responses to multi-level warming and supplemental summer rain in a high Arctic ecosystem. Global Change Biology.

[b89] Sistla SA, Moore JC, Simpson RT (2013). Long-term warming restructures Arctic tundra without changing net soil carbon storage. Nature.

[b90] Strand AE, Pritchard SG, McCormack ML, Davis MA, Oren R (2008). Irreconcilable differences: fine-root life spans and soil carbon persistence. Science.

[b91] Sturm M, McFadden JP, Liston GE, Chapin FS, Racine CH, Holmgren J (2001). Snow – shrub interactions in Arctic Tundra: a hypothesis with climatic implications. Journal of Climate.

[b92] Sturm M, Douglas T, Racine C, Liston G (2005). Changing snow and shrub conditions affect albedo with global implications. Journal of Geophysical Research.

[b93] Sullivan PF, Welker JM (2005). Warming chambers stimulate early season growth of an Arctic sedge: results of a minirhizotron field study. Oecologia.

[b94] Sullivan PF, Sommerkorn M, Rueth H, Nadelhoffer K, Shaver G, Welker JM (2007). Climate and species affect fine root production with long-term fertilization in acidic tussock tundra near Toolik Lake, Alaska. Oecologia.

[b95] Talbot JM, Bruns TD, Smith DP (2013). Independent roles of ectomycorrhizal and saprotrophic communities in soil organic matter decomposition. Soil Biology and Biochemistry.

[b96] Tape K, Sturm M, Racine C (2006). The evidence for shrub expansion in Northern Alaska and the Pan-Arctic. Global Change Biology.

[b97] Tape KD, Hallinger M, Welker JM, Ruess RW (2012). Landscape heterogeneity of shrub expansion in Arctic Alaska. Ecosystems.

[b98] Tarnocai C, Canadell JG, Schuur E aG, Kuhry P, Mazhitova G, Zimov S (2009). Soil organic carbon pools in the northern circumpolar permafrost region. Global Biogeochemical Cycles.

[b99] Tedersoo L, Smith ME (2013). Lineages of ectomycorrhizal fungi revisited: foraging strategies and novel lineages revealed by sequences from belowground. Fungal Biology Reviews.

[b100] Tedersoo L, May TW, Smith ME (2010). Ectomycorrhizal lifestyle in fungi: global diversity, distribution, and evolution of phylogenetic lineages. Mycorrhiza.

[b101] Tedersoo L, Naadel T, Bahram M (2012). Enzymatic activities and stable isotope patterns of ectomycorrhizal fungi in relation to phylogeny and exploration types in an afrotropical rain forest. New Phytologist.

[b102] Timling I, Taylor DL (2012). Peeking through a frosty window: molecular insights into the ecology of Arctic soil fungi. Fungal Ecology.

[b103] Timling I, Dahlberg A, Walker DA, Gardes M, Charcosset JY, Welker JM, Taylor DL (2012). Distribution and drivers of ectomycorrhizal fungal communities across the North American Arctic. Ecosphere.

[b104] Timling I, Walker DA, Nusbaum C (2014). Rich and cold: diversity, distribution and drivers of fungal communities in patterned-ground ecosystems of the North American Arctic. Molecular Ecology.

[b105] Toljander JF, Eberhardt U, Toljander YK, Paul LR, Taylor AFS (2006). Species composition of an ectomycorrhizal fungal community along a local nutrient gradient in a boreal forest. New Phytologist.

[b106] Twieg BD, Durall DM, Simard SW, Jones MD (2009). Influence of soil nutrients on ectomycorrhizal communities in a chronosequence of mixed temperate forests. Mycorrhiza.

[b107] Unestam T, Sun Y (1995). Extramatrical structures of hydrophobic and hydrophilic ectomycorrhizal fungi. Mycorrhiza.

[b108] Urban A, Weib M, Bauer R (2003). Ectomycorrhizas involving sebacinoid mycobionts. Mycological Research.

[b109] Wahren CHA, Walker MD, Bret-Harte MS (2005). Vegetation responses in Alaskan arctic tundra after 8 years of a summer warming and winter snow manipulation experiment. Global Change Biology.

[b110] Walker MD, Walker DA, Welker JM (1999). Long-term experimental manipulation of winter snow regime and summer temperature in arctic and alpine tundra. Hydrological Processes.

[b111] Walker DA, Raynolds MK, Daniëls FJA (2005). The circumpolar Arctic vegetation map. Journal of Vegetation Science.

[b112] Weigt RB, Raidl S, Verma R, Agerer R (2011). Exploration type-specific standard values of extramatrical mycelium – a step towards quantifying ectomycorrhizal space occupation and biomass in natural soil. Mycological Progress.

[b113] Weintraub MN, Schimel JP (2005). The seasonal dynamics of amino acids and other nutrients in Alaskan Arctic tundra soils. Biogeochemistry.

[b114] Welker JM, Molau U, Parsons AN, Robinson CH, Wookey PA (1997). Response of *Dryas octopetala* to ITEX manipulations: a synthesis with circumpolar comparisons. Global Change Biology.

[b115] Welker JM, Fahnestock JT, Jones MH (2000). Annual CO2 flux from dry and moist acidic tundra: field responses to increases in summer temperature and winter snow depth. Climatic Change.

[b116] Welker JM, Fahnestock JT, Henry GHR, O'Dea KW, Chimner RA (2004). CO2 exchange in three Canadian High Arctic ecosystems: response to long-term experimental warming. Global Change Biology.

[b117] Welker JM, Fahnestock JT, Sullivan PF, Chimner RA (2005). Leaf mineral nutrition of arctic plants in response to long-term warming and deeper snow in N. Alaska. Oikos.

[b118] White TM, Bruns T, Lee S, Taylor J, Innis MA, Gelfand DH, Sninsky JJ, White TJ (1990). Amplification and direct sequencing of fungal ribosomal RNA for phylogenetics. PCR Protocols: A Guide to Methods and Applications.

[b119] Wright DP, Johansson T, Le Quéré A, Söderström B, Tunlid A (2005). Spatial patterns of gene expression in the extramatrical mycelium and mycorrhizal root tips formed by the ectomycorrhizal fungus *Paxillus involutus* in association with birch (*Betula pendula*) seedlings in soil microcosms. New Phytologist.

[b1004] Zhang W, Miller PA, Smith B, Wania R, Koenigk T, Döscher R (2013). Tundra shrubification and tree-line advance amplify arctic climate warming: results from an individual-based dynamic vegetation model. Environmental Research Letters.

